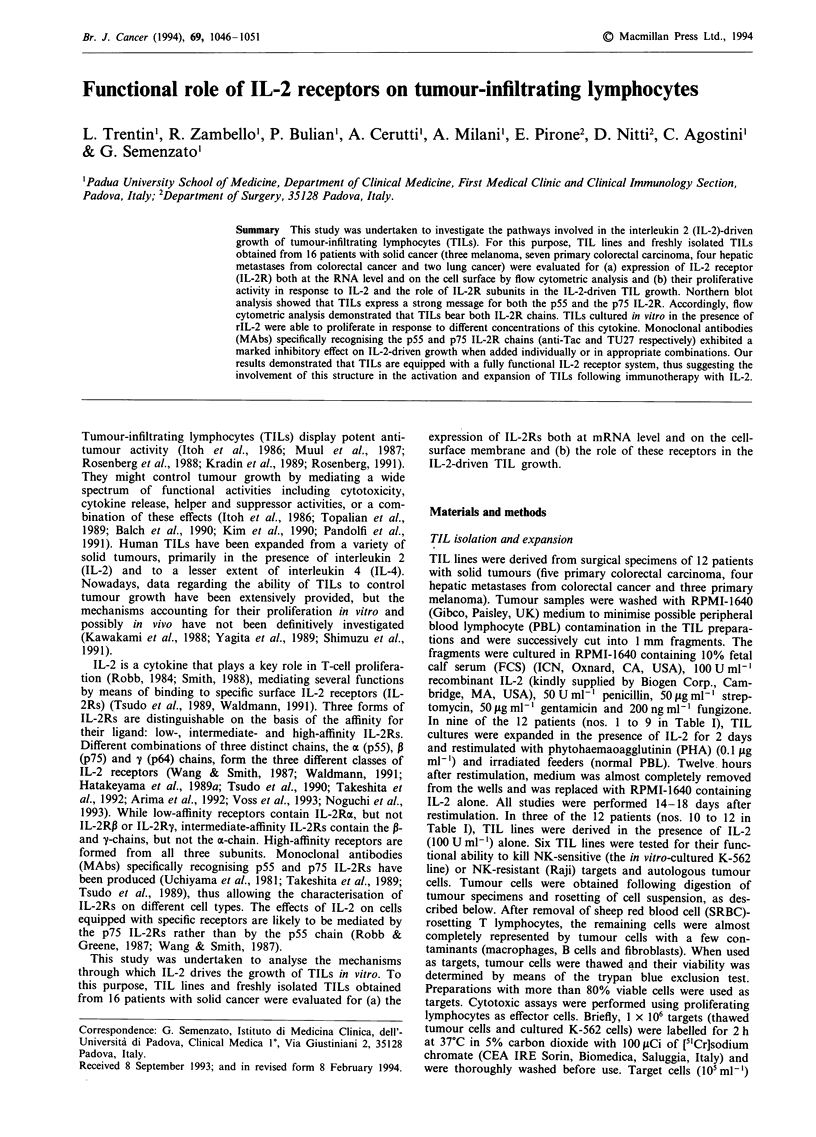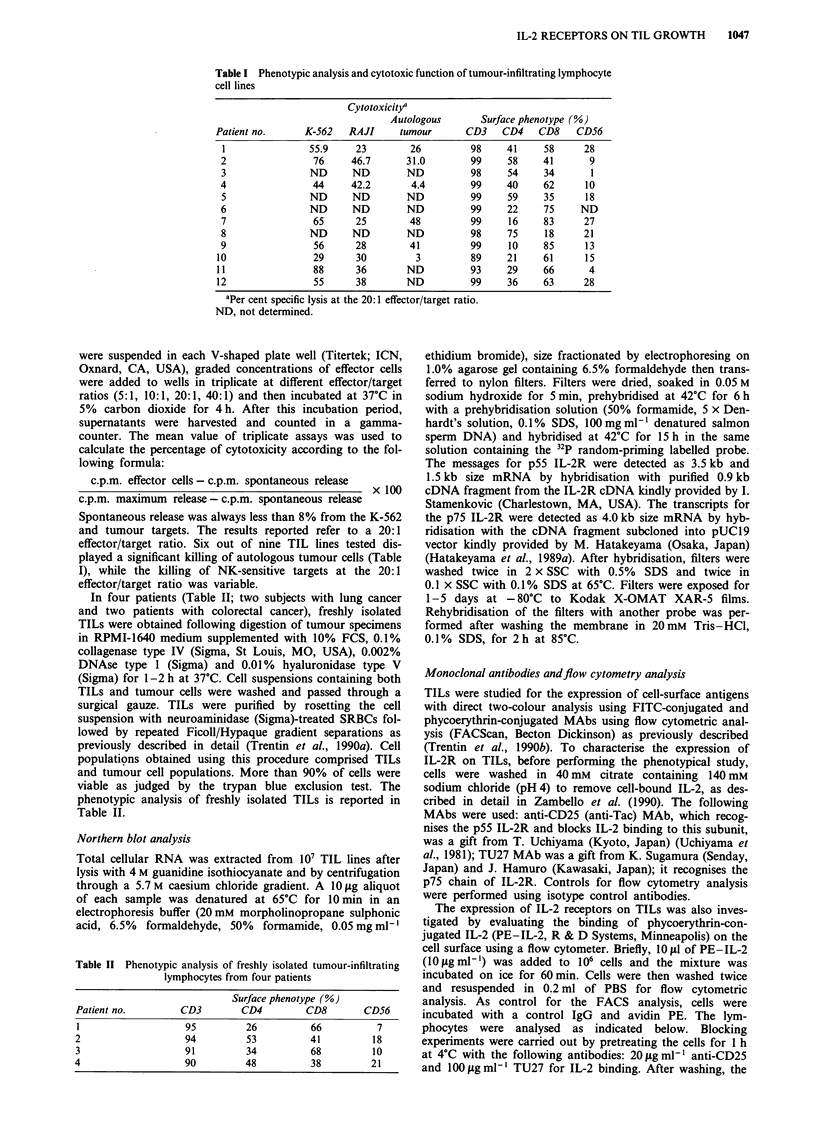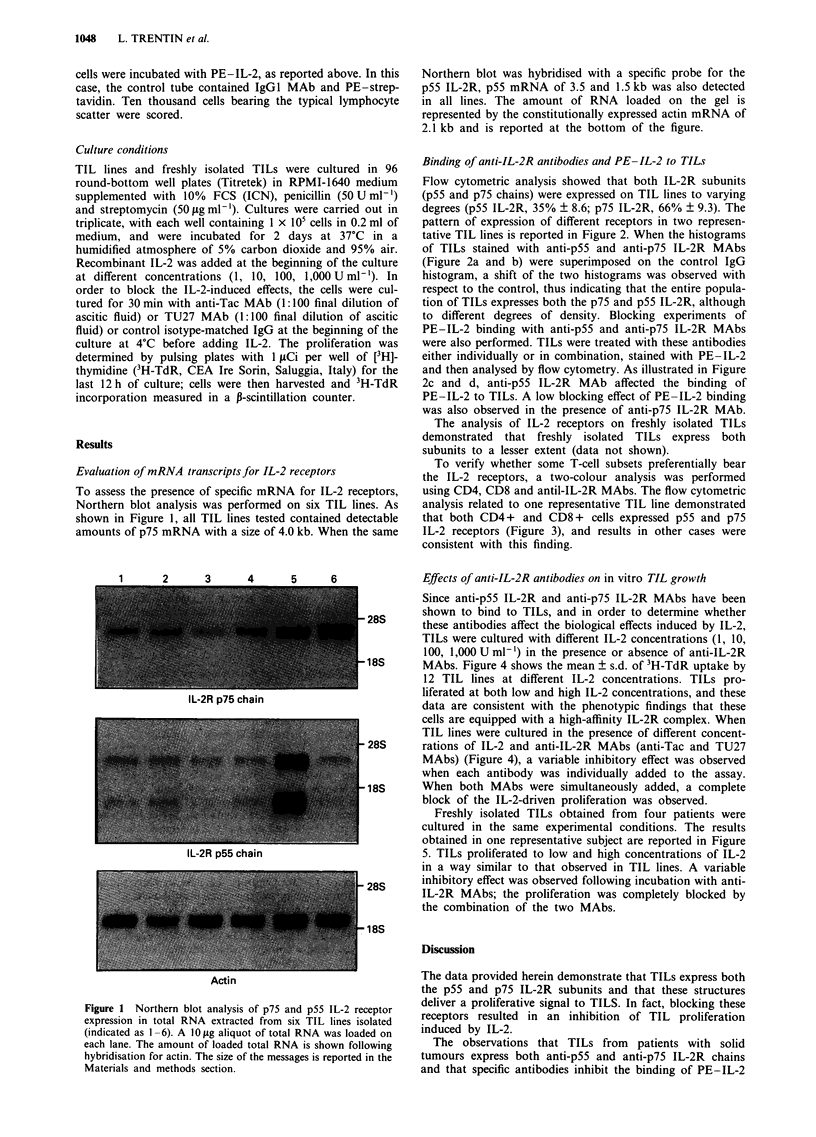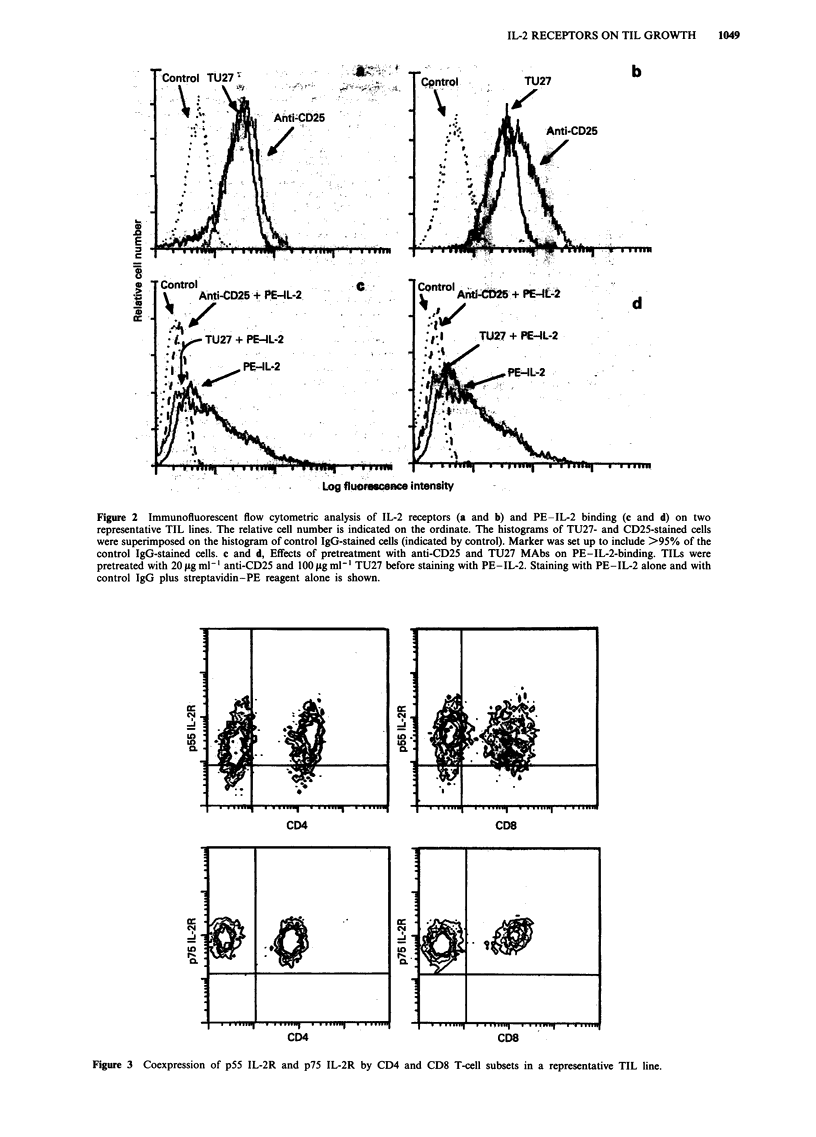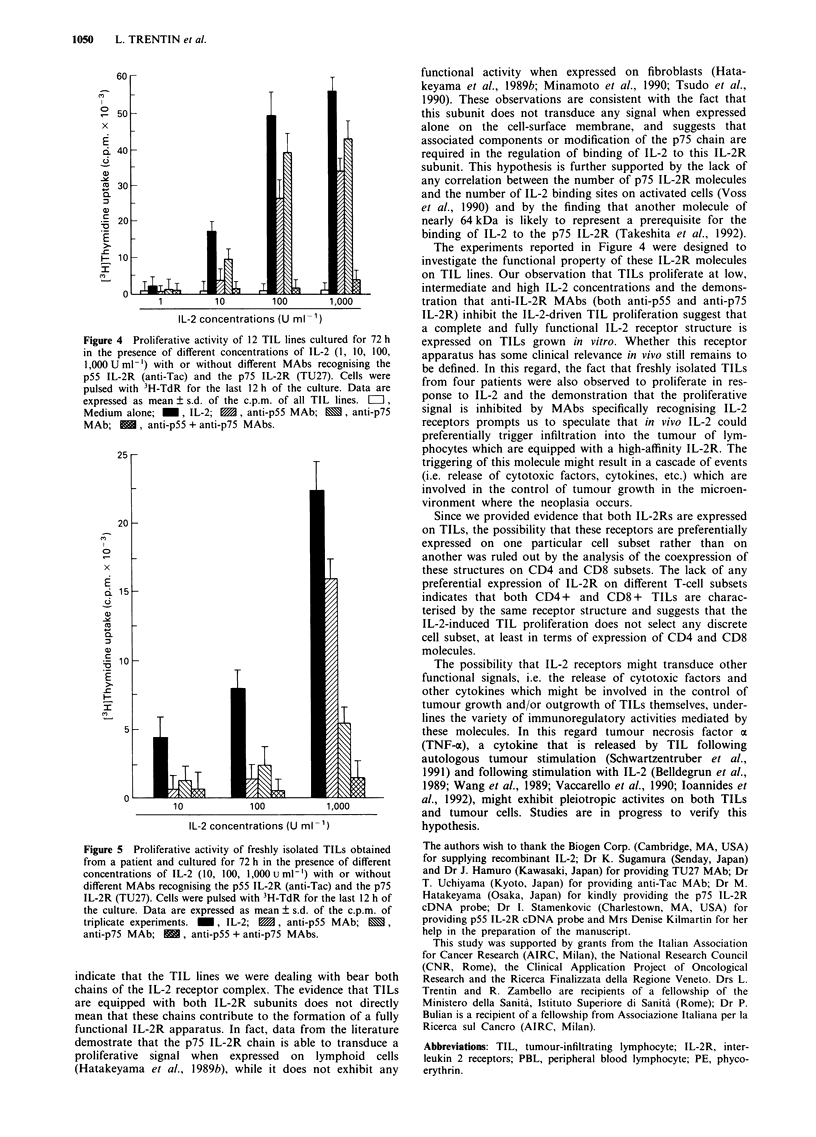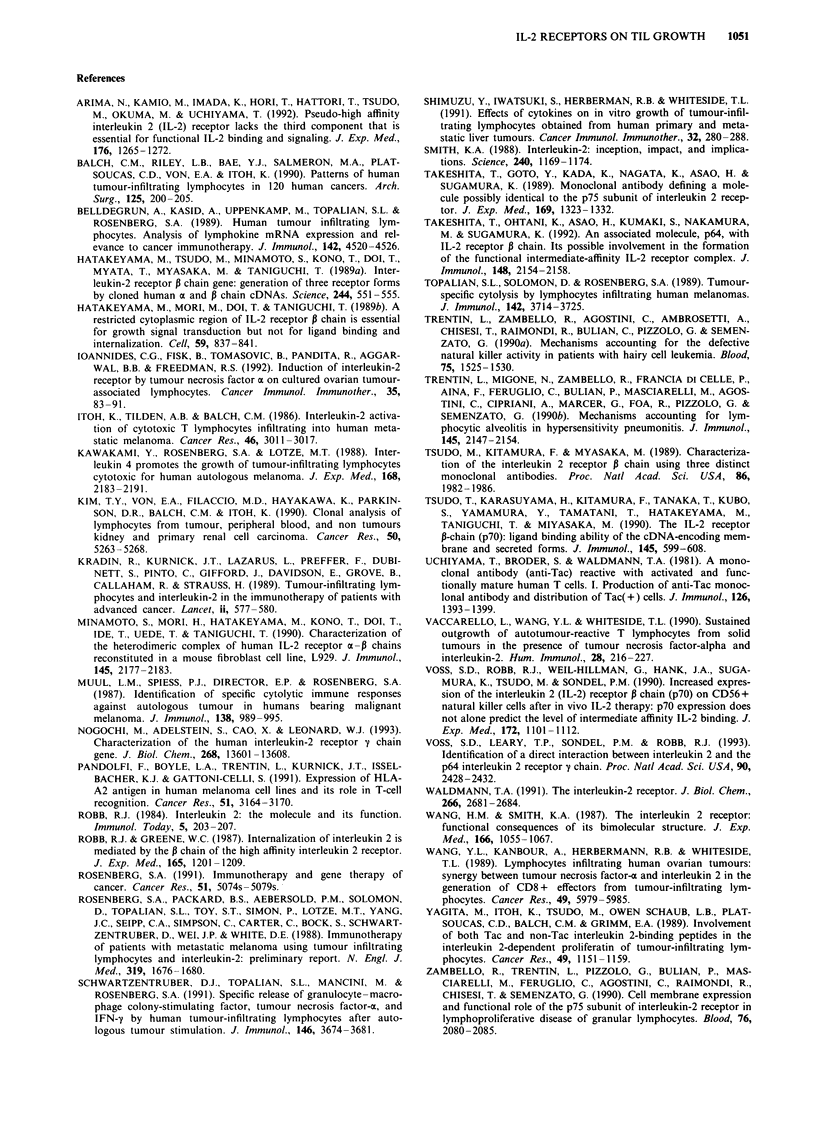# Functional role of IL-2 receptors on tumour-infiltrating lymphocytes.

**DOI:** 10.1038/bjc.1994.206

**Published:** 1994-06

**Authors:** L. Trentin, R. Zambello, P. Bulian, A. Cerutti, A. Milani, E. Pirone, D. Nitti, C. Agostini, G. Semenzato

**Affiliations:** Padua University School of Medicine, Department of Clinical Medicine, Padova, Italy.

## Abstract

**Images:**


Br. J. Cancer (1994), 69, 1046-1051 © Macmillan Press Ltd., 1994

### Functional role of IL-2 receptors on tumour-infiltrating lymphocytes

L. Trentin[1], R. Zambello[1], P. Bulian[1], A. Cerutti[1], A. Milani[1], E. Pirone[2], D. Nitti[2], C. Agostini[1] & G. Semenzato[1]

[1]*Padua University School of Medicine, Department of Clinical Medicine, First Medical Clinic and Clinical Immunology Section, Padova, Italy;* [2]*Department of Surgery, 35128 Padova, Italy.*

**Summary** This study was undertaken to investigate the pathways involved in the interleukin 2 (IL-2)-driven growth of tumour-infiltrating lymphocytes (TILs). For this purpose, TIL lines and freshly isolated TILs obtained from 16 patients with solid cancer (three melanoma, seven primary colorectal carcinoma, four hepatic metastases from colorectal cancer and two lung cancer) were evaluated for (a) expression of IL-2 receptor (IL-2R) both at the RNA level and on the cell surface by flow cytometric analysis and (b) their proliferative activity in response to IL-2 and the role of IL-2R subunits in the IL-2-driven TIL growth. Northern blot analysis showed that TILs express a strong message for both the p55 and the p75 IL-2R. Accordingly, flow cytometric analysis demonstrated that TILs bear both IL-2R chains. TILs cultured *in vitro* in the presence of rIL-2 were able to proliferate in response to different concentrations of this cytokine. Monoclonal antibodies (MAbs) specifically recognising the p55 and p75 IL-2R chains (anti-Tac and TU27 respectively) exhibited a marked inhibitory effect on IL-2-driven growth when added individually or in appropriate combinations. Our results demonstrated that TILs are equipped with a fully functional IL-2 receptor system, thus suggesting the involvement of this structure in the activation and expansion of TILs following immunotherapy with IL-2.

Tumour-infiltrating lymphocytes (TILs) display potent anti-tumour activity (Itoh *et al.*, 1986; Muul *et al.*, 1987; Rosenberg *et al.*, 1988; Kradin *et al.*, 1989; Rosenberg, 1991). They might control tumour growth by mediating a wide spectrum of functional activities including cytotoxicity, cytokine release, helper and suppressor activities, or a combination of these effects (Itoh *et al.*, 1986; Topalian *et al.*, 1989; Balch *et al.*, 1990; Kim *et al.*, 1990; Pandolfi *et al.*, 1991). Human TILs have been expanded from a variety of solid tumours, primarily in the presence of interleukin 2 (IL-2) and to a lesser extent of interleukin 4 (IL-4). Nowadays, data regarding the ability of TILs to control tumour growth have been extensively provided, but the mechanisms accounting for their proliferation *in vitro* and possibly *in vivo* have not been definitively investigated (Kawakami *et al.*, 1988; Yagita *et al.*, 1989; Shimuzu *et al.*, 1991).

IL-2 is a cytokine that plays a key role in T-cell proliferation (Robb, 1984; Smith, 1988), mediating several functions by means of binding to specific surface IL-2 receptors (IL-2Rs) (Tsudo *et al.*, 1989, Waldmann, 1991). Three forms of IL-2Rs are distinguishable on the basis of the affinity for their ligand: low-, intermediate- and high-affinity IL-2Rs. Different combinations of three distinct chains, the $\alpha$ (p55), $\beta$ (p75) and $\gamma$ (p64) chains, form the three different classes of IL-2 receptors (Wang & Smith, 1987; Waldmann, 1991; Hatakeyama *et al.*, 1989*a*; Tsudo *et al.*, 1990; Takeshita *et al.*, 1992; Arima *et al.*, 1992; Voss *et al.*, 1993; Noguchi *et al.*, 1993). While low-affinity receptors contain IL-2R$\alpha$, but not IL-2R$\beta$ or IL-2R$\gamma$, intermediate-affinity IL-2Rs contain the $\beta$- and $\gamma$-chains, but not the $\alpha$-chain. High-affinity receptors are formed from all three subunits. Monoclonal antibodies (MAbs) specifically recognising p55 and p75 IL-2Rs have been produced (Uchiyama *et al.*, 1981; Takeshita *et al.*, 1989; Tsudo *et al.*, 1989), thus allowing the characterisation of IL-2Rs on different cell types. The effects of IL-2 on cells equipped with specific receptors are likely to be mediated by the p75 IL-2Rs rather than by the p55 chain (Robb & Greene, 1987; Wang & Smith, 1987).

This study was undertaken to analyse the mechanisms through which IL-2 drives the growth of TILs *in vitro*. To this purpose, TIL lines and freshly isolated TILs obtained from 16 patients with solid cancer were evaluated for (a) the expression of IL-2Rs both at mRNA level and on the cell-surface membrane and (b) the role of these receptors in the IL-2-driven TIL growth.

Correspondence: G. Semenzato, Istituto di Medicina Clinica, dell'Università di Padova, Clinical Medica 1°, Via Giustiniani 2, 35128 Padova, Italy.
Received 8 September 1993; and in revised form 8 February 1994.

### Materials and methods

### *TIL isolation and expansion*

TIL lines were derived from surgical specimens of 12 patients with solid tumours (five primary colorectal carcinoma, four hepatic metastases from colorectal cancer and three primary melanoma). Tumour samples were washed with RPMI-1640 (Gibco, Paisley, UK) medium to minimise possible peripheral blood lymphocyte (PBL) contamination in the TIL preparations and were successively cut into 1 mm fragments. The fragments were cultured in RPMI-1640 containing 10% fetal calf serum (FCS) (ICN, Oxnard, CA, USA), $100\ U\ ml^{-1}$ recombinant IL-2 (kindly supplied by Biogen Corp., Cambridge, MA, USA), $50\ U\ ml^{-1}$ penicillin, $50\ \mu g\ ml^{-1}$ streptomycin, $50\ \mu g\ ml^{-1}$ gentamicin and $200\ ng\ ml^{-1}$ fungizone. In nine of the 12 patients (nos. 1 to 9 in Table I), TIL cultures were expanded in the presence of IL-2 for 2 days and restimulated with phytohaemaoagglutinin (PHA) ($0.1\ \mu g\ ml^{-1}$) and irradiated feeders (normal PBL). Twelve hours after restimulation, medium was almost completely removed from the wells and was replaced with RPMI-1640 containing IL-2 alone. All studies were performed 14-18 days after restimulation. In three of the 12 patients (nos. 10 to 12 in Table I), TIL lines were derived in the presence of IL-2 ($100\ U\ ml^{-1}$) alone. Six TIL lines were tested for their functional ability to kill NK-sensitive (the *in vitro*-cultured K-562 line) or NK-resistant (Raji) targets and autologous tumour cells. Tumour cells were obtained following digestion of tumour specimens and rosetting of cell suspension, as described below. After removal of sheep red blood cell (SRBC)-rosetting T lymphocytes, the remaining cells were almost completely represented by tumour cells with a few contaminants (macrophages, B cells and fibroblasts). When used as targets, tumour cells were thawed and their viability was determined by means of the trypan blue exclusion test. Preparations with more than 80% viable cells were used as targets. Cytotoxic assays were performed using proliferating lymphocytes as effector cells. Briefly, $1 \times 10^6$ targets (thawed tumour cells and cultured K-562 cells) were labelled for 2 h at 37°C in 5% carbon dioxide with $100\ \mu Ci$ of [$^{51}Cr$]sodium chromate (CEA IRE Sorin, Biomedica, Saluggia, Italy) and were thoroughly washed before use. Target cells ($10^5\ ml^{-1}$)

**Table I** Phenotypic analysis and cytotoxic function of tumour-infiltrating lymphocyte cell lines

| | Cytotoxicity[a] | | | Surface phenotype (%) | | | |
|---|---|---|---|---|---|---|---|
| *Patient no.* | *K-562* | *RAJI* | *Autologous tumour* | *CD3* | *CD4* | *CD8* | *CD56* |
| 1 | 55.9 | 23 | 26 | 98 | 41 | 58 | 28 |
| 2 | 76 | 46.7 | 31.0 | 99 | 58 | 41 | 9 |
| 3 | ND | ND | ND | 98 | 54 | 34 | 1 |
| 4 | 44 | 42.2 | 4.4 | 99 | 40 | 62 | 10 |
| 5 | ND | ND | ND | 99 | 59 | 35 | 18 |
| 6 | ND | ND | ND | 99 | 22 | 75 | ND |
| 7 | 65 | 25 | 48 | 99 | 16 | 83 | 27 |
| 8 | ND | ND | ND | 98 | 75 | 18 | 21 |
| 9 | 56 | 28 | 41 | 99 | 10 | 85 | 13 |
| 10 | 29 | 30 | 3 | 89 | 21 | 61 | 15 |
| 11 | 88 | 36 | ND | 93 | 29 | 66 | 4 |
| 12 | 55 | 38 | ND | 99 | 36 | 63 | 28 |

[a]Per cent specific lysis at the 20:1 effector/target ratio.
ND, not determined.

were suspended in each V-shaped plate well (Titertek; ICN, Oxnard, CA, USA), graded concentrations of effector cells were added to wells in triplicate at different effector/target ratios (5:1, 10:1, 20:1, 40:1) and then incubated at 37°C in 5% carbon dioxide for 4 h. After this incubation period, supernatants were harvested and counted in a gamma-counter. The mean value of triplicate assays was used to calculate the percentage of cytotoxicity according to the following formula:

$$\frac{\text{c.p.m. effector cells} - \text{c.p.m. spontaneous release}}{\text{c.p.m. maximum release} - \text{c.p.m. spontaneous release}} \times 100$$

Spontaneous release was always less than 8% from the K-562 and tumour targets. The results reported refer to a 20:1 effector/target ratio. Six out of nine TIL lines tested displayed a significant killing of autologous tumour cells (Table I), while the killing of NK-sensitive targets at the 20:1 effector/target ratio was variable.

In four patients (Table II; two subjects with lung cancer and two patients with colorectal cancer), freshly isolated TILs were obtained following digestion of tumour specimens in RPMI-1640 medium supplemented with 10% FCS, 0.1% collagenase type IV (Sigma, St Louis, MO, USA), 0.002% DNAse type 1 (Sigma) and 0.01% hyaluronidase type V (Sigma) for 1–2 h at 37°C. Cell suspensions containing both TILs and tumour cells were washed and passed through a surgical gauze. TILs were purified by rosetting the cell suspension with neuroaminidase (Sigma)-treated SRBCs followed by repeated Ficoll/Hypaque gradient separations as previously described in detail (Trentin *et al.*, 1990*a*). Cell populations obtained using this procedure comprised TILs and tumour cell populations. More than 90% of cells were viable as judged by the trypan blue exclusion test. The phenotypic analysis of freshly isolated TILs is reported in Table II.

*Northern blot analysis*

Total cellular RNA was extracted from $10^7$ TIL lines after lysis with 4 M guanidine isothiocyanate and by centrifugation through a 5.7 M caesium chloride gradient. A 10 μg aliquot of each sample was denatured at 65°C for 10 min in an electrophoresis buffer (20 mM morpholinopropane sulphonic acid, 6.5% formaldehyde, 50% formamide, 0.05 mg ml$^{-1}$ ethidium bromide), size fractionated by electrophoresing on 1.0% agarose gel containing 6.5% formaldehyde then transferred to nylon filters. Filters were dried, soaked in 0.05 M sodium hydroxide for 5 min, prehybridised at 42°C for 6 h with a prehybridisation solution (50% formamide, 5 × Denhardt's solution, 0.1% SDS, 100 mg ml$^{-1}$ denatured salmon sperm DNA) and hybridised at 42°C for 15 h in the same solution containing the $^{32}P$ random-priming labelled probe. The messages for p55 IL-2R were detected as 3.5 kb and 1.5 kb size mRNA by hybridisation with purified 0.9 kb cDNA fragment from the IL-2R cDNA kindly provided by I. Stamenkovic (Charlestown, MA, USA). The transcripts for the p75 IL-2R were detected as 4.0 kb size mRNA by hybridisation with the cDNA fragment subcloned into pUC19 vector kindly provided by M. Hatakeyama (Osaka, Japan) (Hatakeyama *et al.*, 1989*a*). After hybridisation, filters were washed twice in 2 × SSC with 0.5% SDS and twice in 0.1 × SSC with 0.1% SDS at 65°C. Filters were exposed for 1–5 days at −80°C to Kodak X-OMAT XAR-5 films. Rehybridisation of the filters with another probe was performed after washing the membrane in 20 mM Tris–HCl, 0.1% SDS, for 2 h at 85°C.

**Table II** Phenotypic analysis of freshly isolated tumour-infiltrating lymphocytes from four patients

| | Surface phenotype (%) | | | |
|---|---|---|---|---|
| *Patient no.* | *CD3* | *CD4* | *CD8* | *CD56* |
| 1 | 95 | 26 | 66 | 7 |
| 2 | 94 | 53 | 41 | 18 |
| 3 | 91 | 34 | 68 | 10 |
| 4 | 90 | 48 | 38 | 21 |

*Monoclonal antibodies and flow cytometry analysis*

TILs were studied for the expression of cell-surface antigens with direct two-colour analysis using FITC-conjugated and phycoerythrin-conjugated MAbs using flow cytometric analysis (FACScan, Becton Dickinson) as previously described (Trentin *et al.*, 1990*b*). To characterise the expression of IL-2R on TILs, before performing the phenotypical study, cells were washed in 40 mM citrate containing 140 mM sodium chloride (pH 4) to remove cell-bound IL-2, as described in detail in Zambello *et al.* (1990). The following MAbs were used: anti-CD25 (anti-Tac) MAb, which recognises the p55 IL-2R and blocks IL-2 binding to this subunit, was a gift from T. Uchiyama (Kyoto, Japan) (Uchiyama *et al.*, 1981); TU27 MAb was a gift from K. Sugamura (Senday, Japan) and J. Hamuro (Kawasaki, Japan); it recognises the p75 chain of IL-2R. Controls for flow cytometry analysis were performed using isotype control antibodies.

The expression of IL-2 receptors on TILs was also investigated by evaluating the binding of phycoerythrin-conjugated IL-2 (PE–IL-2, R & D Systems, Minneapolis) on the cell surface using a flow cytometer. Briefly, 10 μl of PE–IL-2 (10 μg ml$^{-1}$) was added to $10^6$ cells and the mixture was incubated on ice for 60 min. Cells were then washed twice and resuspended in 0.2 ml of PBS for flow cytometric analysis. As control for the FACS analysis, cells were incubated with a control IgG and avidin PE. The lymphocytes were analysed as indicated below. Blocking experiments were carried out by pretreating the cells for 1 h at 4°C with the following antibodies: 20 μg ml$^{-1}$ anti-CD25 and 100 μg ml$^{-1}$ TU27 for IL-2 binding. After washing, the cells were incubated with PE–IL-2, as reported above. In this case, the control tube contained IgG1 MAb and PE–streptavidin. Ten thousand cells bearing the typical lymphocyte scatter were scored.

*Culture conditions*

TIL lines and freshly isolated TILs were cultured in 96 round-bottom well plates (Titretek) in RPMI-1640 medium supplemented with 10% FCS (ICN), penicillin (50 U $ml^{-1}$) and streptomycin (50 $\mu g$ $ml^{-1}$). Cultures were carried out in triplicate, with each well containing $1 \times 10^5$ cells in 0.2 ml of medium, and were incubated for 2 days at 37°C in a humidified atmosphere of 5% carbon dioxide and 95% air. Recombinant IL-2 was added at the beginning of the culture at different concentrations (1, 10, 100, 1,000 U $ml^{-1}$). In order to block the IL-2-induced effects, the cells were cultured for 30 min with anti-Tac MAb (1:100 final dilution of ascitic fluid) or TU27 MAb (1:100 final dilution of ascitic fluid) or control isotype-matched IgG at the beginning of the culture at 4°C before adding IL-2. The proliferation was determined by pulsing plates with 1 $\mu$Ci per well of [$^3$H]-thymidine ($^3$H-TdR, CEA Ire Sorin, Saluggia, Italy) for the last 12 h of culture; cells were then harvested and $^3$H-TdR incorporation measured in a $\beta$-scintillation counter.

### Results

*Evaluation of mRNA transcripts for IL-2 receptors*

To assess the presence of specific mRNA for IL-2 receptors, Northern blot analysis was performed on six TIL lines. As shown in Figure 1, all TIL lines tested contained detectable amounts of p75 mRNA with a size of 4.0 kb. When the same Northern blot was hybridised with a specific probe for the p55 IL-2R, p55 mRNA of 3.5 and 1.5 kb was also detected in all lines. The amount of RNA loaded on the gel is represented by the constitutionally expressed actin mRNA of 2.1 kb and is reported at the bottom of the figure.

**Figure 1** Northern blot analysis of p75 and p55 IL-2 receptor expression in total RNA extracted from six TIL lines isolated (indicated as 1–6). A 10 $\mu$g aliquot of total RNA was loaded on each lane. The amount of loaded total RNA is shown following hybridisation for actin. The size of the messages is reported in the Materials and methods section.

*Binding of anti-IL-2R antibodies and PE–IL-2 to TILs*

Flow cytometric analysis showed that both IL-2R subunits (p55 and p75 chains) were expressed on TIL lines to varying degrees (p55 IL-2R, 35% ± 8.6; p75 IL-2R, 66% ± 9.3). The pattern of expression of different receptors in two representative TIL lines is reported in Figure 2. When the histograms of TILs stained with anti-p55 and anti-p75 IL-2R MAbs (Figure 2a and b) were superimposed on the control IgG histogram, a shift of the two histograms was observed with respect to the control, thus indicating that the entire population of TILs expresses both the p75 and p55 IL-2R, although to different degrees of density. Blocking experiments of PE–IL-2 binding with anti-p55 and anti-p75 IL-2R MAbs were also performed. TILs were treated with these antibodies either individually or in combination, stained with PE–IL-2 and then analysed by flow cytometry. As illustrated in Figure 2c and d, anti-p55 IL-2R MAb affected the binding of PE–IL-2 to TILs. A low blocking effect of PE–IL-2 binding was also observed in the presence of anti-p75 IL-2R MAb.

The analysis of IL-2 receptors on freshly isolated TILs demonstrated that freshly isolated TILs express both subunits to a lesser extent (data not shown).

To verify whether some T-cell subsets preferentially bear the IL-2 receptors, a two-colour analysis was performed using CD4, CD8 and antil-IL-2R MAbs. The flow cytometric analysis related to one representative TIL line demonstrated that both CD4+ and CD8+ cells expressed p55 and p75 IL-2 receptors (Figure 3), and results in other cases were consistent with this finding.

*Effects of anti-IL-2R antibodies on* in vitro *TIL growth*

Since anti-p55 IL-2R and anti-p75 IL-2R MAbs have been shown to bind to TILs, and in order to determine whether these antibodies affect the biological effects induced by IL-2, TILs were cultured with different IL-2 concentrations (1, 10, 100, 1,000 U $ml^{-1}$) in the presence or absence of anti-IL-2R MAbs. Figure 4 shows the mean ± s.d. of $^3$H-TdR uptake by 12 TIL lines at different IL-2 concentrations. TILs proliferated at both low and high IL-2 concentrations, and these data are consistent with the phenotypic findings that these cells are equipped with a high-affinity IL-2R complex. When TIL lines were cultured in the presence of different concentrations of IL-2 and anti-IL-2R MAbs (anti-Tac and TU27 MAbs) (Figure 4), a variable inhibitory effect was observed when each antibody was individually added to the assay. When both MAbs were simultaneously added, a complete block of the IL-2-driven proliferation was observed.

Freshly isolated TILs obtained from four patients were cultured in the same experimental conditions. The results obtained in one representative subject are reported in Figure 5. TILs proliferated to low and high concentrations of IL-2 in a way similar to that observed in TIL lines. A variable inhibitory effect was observed following incubation with anti-IL-2R MAbs; the proliferation was completely blocked by the combination of the two MAbs.

### Discussion

The data provided herein demonstrate that TILs express both the p55 and p75 IL-2R subunits and that these structures deliver a proliferative signal to TILS. In fact, blocking these receptors resulted in an inhibition of TIL proliferation induced by IL-2.

The observations that TILs from patients with solid tumours express both anti-p55 and anti-p75 IL-2R chains and that specific antibodies inhibit the binding of PE–IL-2

**Figure 2** Immunofluorescent flow cytometric analysis of IL-2 receptors (**a** and **b**) and PE–IL-2 binding (**c** and **d**) on two representative TIL lines. The relative cell number is indicated on the ordinate. The histograms of TU27- and CD25-stained cells were superimposed on the histogram of control IgG-stained cells (indicated by control). Marker was set up to include >95% of the control IgG-stained cells. **c** and **d**, Effects of pretreatment with anti-CD25 and TU27 MAbs on PE–IL-2-binding. TILs were pretreated with 20 $\mu g\ ml^{-1}$ anti-CD25 and 100 $\mu g\ ml^{-1}$ TU27 before staining with PE–IL-2. Staining with PE–IL-2 alone and with control IgG plus streptavidin–PE reagent alone is shown.

**Figure 3** Coexpression of p55 IL-2R and p75 IL-2R by CD4 and CD8 T-cell subsets in a representative TIL line.

**Figure 4** Proliferative activity of 12 TIL lines cultured for 72 h in the presence of different concentrations of IL-2 (1, 10, 100, 1,000 U $ml^{-1}$) with or without different MAbs recognising the p55 IL-2R (anti-Tac) and the p75 IL-2R (TU27). Cells were pulsed with $^{3}$H-TdR for the last 12 h of the culture. Data are expressed as mean ± s.d. of the c.p.m. of all TIL lines. □, Medium alone; ■, IL-2; ▨, anti-p55 MAb; ▧, anti-p75 MAb; ▩, anti-p55 + anti-p75 MAbs.

**Figure 5** Proliferative activity of freshly isolated TILs obtained from a patient and cultured for 72 h in the presence of different concentrations of IL-2 (10, 100, 1,000 U $ml^{-1}$) with or without different MAbs recognising the p55 IL-2R (anti-Tac) and the p75 IL-2R (TU27). Cells were pulsed with $^{3}$H-TdR for the last 12 h of the culture. Data are expressed as mean ± s.d. of the c.p.m. of triplicate experiments. ■, IL-2; ▨, anti-p55 MAb; ▧, anti-p75 MAb; ▩, anti-p55 + anti-p75 MAbs.

indicate that the TIL lines we were dealing with bear both chains of the IL-2 receptor complex. The evidence that TILs are equipped with both IL-2R subunits does not directly mean that these chains contribute to the formation of a fully functional IL-2R apparatus. In fact, data from the literature demostrate that the p75 IL-2R chain is able to transduce a proliferative signal when expressed on lymphoid cells (Hatakeyama *et al.*, 1989*b*), while it does not exhibit any functional activity when expressed on fibroblasts (Hatakeyama *et al.*, 1989*b*; Minamoto *et al.*, 1990; Tsudo *et al.*, 1990). These observations are consistent with the fact that this subunit does not transduce any signal when expressed alone on the cell-surface membrane, and suggests that associated components or modification of the p75 chain are required in the regulation of binding of IL-2 to this IL-2R subunit. This hypothesis is further supported by the lack of any correlation between the number of p75 IL-2R molecules and the number of IL-2 binding sites on activated cells (Voss *et al.*, 1990) and by the finding that another molecule of nearly 64 kDa is likely to represent a prerequisite for the binding of IL-2 to the p75 IL-2R (Takeshita *et al.*, 1992).

The experiments reported in Figure 4 were designed to investigate the functional property of these IL-2R molecules on TIL lines. Our observation that TILs proliferate at low, intermediate and high IL-2 concentrations and the demonstration that anti-IL-2R MAbs (both anti-p55 and anti-p75 IL-2R) inhibit the IL-2-driven TIL proliferation suggest that a complete and fully functional IL-2 receptor structure is expressed on TILs grown *in vitro*. Whether this receptor apparatus has some clinical relevance *in vivo* still remains to be defined. In this regard, the fact that freshly isolated TILs from four patients were also observed to proliferate in response to IL-2 and the demonstration that the proliferative signal is inhibited by MAbs specifically recognising IL-2 receptors prompts us to speculate that *in vivo* IL-2 could preferentially trigger infiltration into the tumour of lymphocytes which are equipped with a high-affinity IL-2R. The triggering of this molecule might result in a cascade of events (i.e. release of cytotoxic factors, cytokines, etc.) which are involved in the control of tumour growth in the microenvironment where the neoplasia occurs.

Since we provided evidence that both IL-2Rs are expressed on TILs, the possibility that these receptors are preferentially expressed on one particular cell subset rather than on another was ruled out by the analysis of the coexpression of these structures on CD4 and CD8 subsets. The lack of any preferential expression of IL-2R on different T-cell subsets indicates that both CD4+ and CD8+ TILs are characterised by the same receptor structure and suggests that the IL-2-induced TIL proliferation does not select any discrete cell subset, at least in terms of expression of CD4 and CD8 molecules.

The possibility that IL-2 receptors might transduce other functional signals, i.e. the release of cytotoxic factors and other cytokines which might be involved in the control of tumour growth and/or outgrowth of TILs themselves, underlines the variety of immunoregulatory activities mediated by these molecules. In this regard tumour necrosis factor α (TNF-α), a cytokine that is released by TIL following autologous tumour stimulation (Schwartzentruber *et al.*, 1991) and following stimulation with IL-2 (Belldegrun *et al.*, 1989; Wang *et al.*, 1989; Vaccarello *et al.*, 1990; Ioannides *et al.*, 1992), might exhibit pleiotropic activites on both TILs and tumour cells. Studies are in progress to verify this hypothesis.

The authors wish to thank the Biogen Corp. (Cambridge, MA, USA) for supplying recombinant IL-2; Dr K. Sugamura (Senday, Japan) and Dr J. Hamuro (Kawasaki, Japan) for providing TU27 MAb; Dr T. Uchiyama (Kyoto, Japan) for providing anti-Tac MAb; Dr M. Hatakeyama (Osaka, Japan) for kindly providing the p75 IL-2R cDNA probe; Dr I. Stamenkovic (Charlestown, MA, USA) for providing p55 IL-2R cDNA probe and Mrs Denise Kilmartin for her help in the preparation of the manuscript.

This study was supported by grants from the Italian Association for Cancer Research (AIRC, Milan), the National Research Council (CNR, Rome), the Clinical Application Project of Oncological Research and the Ricerca Finalizzata della Regione Veneto. Drs L. Trentin and R. Zambello are recipients of a fellowship of the Ministero della Sanità, Istituto Superiore di Sanità (Rome); Dr P. Bulian is a recipient of a fellowship from Associazione Italiana per la Ricerca sul Cancro (AIRC, Milan).

**Abbreviations**: TIL, tumour-infiltrating lymphocyte; IL-2R, interleukin 2 receptors; PBL, peripheral blood lymphocyte; PE, phycoerythrin.

### References

ARIMA, N., KAMIO, M., IMADA, K., HORI, T., HATTORI, T., TSUDO, M., OKUMA, M. & UCHIYAMA, T. (1992). Pseudo-high affinity interleukin 2 (IL-2) receptor lacks the third component that is essential for functional IL-2 binding and signaling. *J. Exp. Med.*, **176**, 1265–1272.

BALCH, C.M., RILEY, L.B., BAE, Y.J., SALMERON, M.A., PLATSOUCAS, C.D., VON, E.A. & ITOH, K. (1990). Patterns of human tumour-infiltrating lymphocytes in 120 human cancers. *Arch. Surg.*, **125**, 200–205.

BELLDEGRUN, A., KASID, A., UPPENKAMP, M., TOPALIAN, S.L. & ROSENBERG, S.A. (1989). Human tumour infiltrating lymphocytes. Analysis of lymphokine mRNA expression and relevance to cancer immunotherapy. *J. Immunol.*, **142**, 4520–4526.

HATAKEYAMA, M., TSUDO, M., MINAMOTO, S., KONO, T., DOI, T., MYATA, T., MYASAKA, M. & TANIGUCHI, T. (1989*a*). Interleukin-2 receptor β chain gene: generation of three receptor forms by cloned human α and β chain cDNAs. *Science*, **244**, 551–555.

HATAKEYAMA, M., MORI, M., DOI, T. & TANIGUCHI, T. (1989*b*). A restricted cytoplasmic region of IL-2 receptor β chain is essential for growth signal transduction but not for ligand binding and internalization. *Cell*, **59**, 837–841.

IOANNIDES, C.G., FISK, B., TOMASOVIC, B., PANDITA, R., AGGARWAL, B.B. & FREEDMAN, R.S. (1992). Induction of interleukin-2 receptor by tumour necrosis factor α on cultured ovarian tumour-associated lymphocytes. *Cancer Immunol. Immunother.*, **35**, 83–91.

ITOH, K., TILDEN, A.B. & BALCH, C.M. (1986). Interleukin-2 activation of cytotoxic T lymphocytes infiltrating into human metastatic melanoma. *Cancer Res.*, **46**, 3011–3017.

KAWAKAMI, Y., ROSENBERG, S.A. & LOTZE, M.T. (1988). Interleukin 4 promotes the growth of tumour-infiltrating lymphocytes cytotoxic for human autologous melanoma. *J. Exp. Med.*, **168**, 2183–2191.

KIM, T.Y., VON, E.A., FILACCIO, M.D., HAYAKAWA, K., PARKINSON, D.R., BALCH, C.M. & ITOH, K. (1990). Clonal analysis of lymphocytes from tumour, peripheral blood, and non tumours kidney and primary renal cell carcinoma. *Cancer Res.*, **50**, 5263–5268.

KRADIN, R., KURNICK, J.T., LAZARUS, L., PREFFER, F., DUBINETT, S., PINTO, C., GIFFORD, J., DAVIDSON, E., GROVE, B., CALLAHAM, R. & STRAUSS, H. (1989). Tumour-infiltrating lymphocytes and interleukin-2 in the immunotherapy of patients with advanced cancer. *Lancet*, **ii**, 577–580.

MINAMOTO, S., MORI, H., HATAKEYAMA, M., KONO, T., DOI, T., IDE, T., UEDE, T. & TANIGUCHI, T. (1990). Characterization of the heterodimeric complex of human IL-2 receptor α–β chains reconstituted in a mouse fibroblast cell line, L929. *J. Immunol.*, **145**, 2177–2183.

MUUL, L.M., SPIESS, P.J., DIRECTOR, E.P. & ROSENBERG, S.A. (1987). Identification of specific cytolytic immune responses against autologous tumour in humans bearing malignant melanoma. *J. Immunol.*, **138**, 989–995.

NOGOCHI, M., ADELSTEIN, S., CAO, X. & LEONARD, W.J. (1993). Characterization of the human interleukin-2 receptor γ chain gene. *J. Biol. Chem.*, **268**, 13601–13608.

PANDOLFI, F., BOYLE, L.A., TRENTIN, L., KURNICK, J.T., ISSELBACHER, K.J. & GATTONI-CELLI, S. (1991). Expression of HLA-A2 antigen in human melanoma cell lines and its role in T-cell recognition. *Cancer Res.*, **51**, 3164–3170.

ROBB, R.J. (1984). Interleukin 2: the molecule and its function. *Immunol. Today*, **5**, 203–207.

ROBB, R.J. & GREENE, W.C. (1987). Internalization of interleukin 2 is mediated by the β chain of the high affinity interleukin 2 receptor. *J. Exp. Med.*, **165**, 1201–1209.

ROSENBERG, S.A. (1991). Immunotherapy and gene therapy of cancer. *Cancer Res.*, **51**, 5074s–5079s.

ROSENBERG, S.A., PACKARD, B.S., AEBERSOLD, P.M., SOLOMON, D., TOPALIAN, S.L., TOY, S.T., SIMON, P., LOTZE, M.T., YANG, J.C., SEIPP, C.A., SIMPSON, C., CARTER, C., BOCK, S., SCHWARTZENTRUBER, D., WEI, J.P. & WHITE, D.E. (1988). Immunotherapy of patients with metastatic melanoma using tumour infiltrating lymphocytes and interleukin-2: preliminary report. *N. Engl. J. Med.*, **319**, 1676–1680.

SCHWARTZENTRUBER, D.J., TOPALIAN, S.L., MANCINI, M. & ROSENBERG, S.A. (1991). Specific release of granulocyte–macrophage colony-stimulating factor, tumour necrosis factor-α, and IFN-γ by human tumour-infiltrating lymphocytes after autologous tumour stimulation. *J. Immunol.*, **146**, 3674–3681.

SHIMUZU, Y., IWATSUKI, S., HERBERMAN, R.B. & WHITESIDE, T.L. (1991). Effects of cytokines on in vitro growth of tumour-infiltrating lymphocytes obtained from human primary and metastatic liver tumours. *Cancer Immunol. Immunother.*, **32**, 280–288.

SMITH, K.A. (1988). Interleukin-2: inception, impact, and implications. *Science*, **240**, 1169–1174.

TAKESHITA, T., GOTO, Y., KADA, K., NAGATA, K., ASAO, H. & SUGAMURA, K. (1989). Monoclonal antibody defining a molecule possibly identical to the p75 subunit of interleukin 2 receptor. *J. Exp. Med.*, **169**, 1323–1332.

TAKESHITA, T., OHTANI, K., ASAO, H., KUMAKI, S., NAKAMURA, M. & SUGAMURA, K. (1992). An associated molecule, p64, with IL-2 receptor β chain. Its possible involvement in the formation of the functional intermediate-affinity IL-2 receptor complex. *J. Immunol.*, **148**, 2154–2158.

TOPALIAN, S.L., SOLOMON, D. & ROSENBERG, S.A. (1989). Tumour-specific cytolysis by lymphocytes infiltrating human melanomas. *J. Immunol.*, **142**, 3714–3725.

TRENTIN, L., ZAMBELLO, R., AGOSTINI, C., AMBROSETTI, A., CHISESI, T., RAIMONDI, R., BULIAN, C., PIZZOLO, G. & SEMENZATO, G. (1990*a*). Mechanisms accounting for the defective natural killer activity in patients with hairy cell leukemia. *Blood*, **75**, 1525–1530.

TRENTIN, L., MIGONE, N., ZAMBELLO, R., FRANCIA DI CELLE, P., AINA, F., FERUGLIO, C., BULIAN, P., MASCIARELLI, M., AGOSTINI, C., CIPRIANI, A., MARCER, G., FOA, R., PIZZOLO, G. & SEMENZATO, G. (1990*b*). Mechanisms accounting for lymphocytic alveolitis in hypersensitivity pneumonitis. *J. Immunol.*, **145**, 2147–2154.

TSUDO, M., KITAMURA, F. & MYASAKA, M. (1989). Characterization of the interleukin 2 receptor β chain using three distinct monoclonal antibodies. *Proc. Natl Acad. Sci. USA*, **86**, 1982–1986.

TSUDO, T., KARASUYAMA, H., KITAMURA, F., TANAKA, T., KUBO, S., YAMAMURA, Y., TAMATANI, T., HATAKEYAMA, M., TANIGUCHI, T. & MIYASAKA, M. (1990). The IL-2 receptor β-chain (p70): ligand binding ability of the cDNA-encoding membrane and secreted forms. *J. Immunol.*, **145**, 599–608.

UCHIYAMA, T., BRODER, S. & WALDMANN, T.A. (1981). A monoclonal antibody (anti-Tac) reactive with activated and functionally mature human T cells. I. Production of anti-Tac monoclonal antibody and distribution of Tac(+) cells. *J. Immunol.*, **126**, 1393–1399.

VACCARELLO, L., WANG, Y.L. & WHITESIDE, T.L. (1990). Sustained outgrowth of autotumour-reactive T lymphocytes from solid tumours in the presence of tumour necrosis factor-alpha and interleukin-2. *Hum. Immunol.*, **28**, 216–227.

VOSS, S.D., ROBB, R.J., WEIL-HILLMAN, G., HANK, J.A., SUGAMURA, K., TSUDO, M. & SONDEL, P.M. (1990). Increased expression of the interleukin 2 (IL-2) receptor β chain (p70) on CD56+ natural killer cells after in vivo IL-2 therapy: p70 expression does not alone predict the level of intermediate affinity IL-2 binding. *J. Exp. Med.*, **172**, 1101–1112.

VOSS, S.D., LEARY, T.P., SONDEL, P.M. & ROBB, R.J. (1993). Identification of a direct interaction between interleukin 2 and the p64 interleukin 2 receptor γ chain. *Proc. Natl Acad. Sci. USA*, **90**, 2428–2432.

WALDMANN, T.A. (1991). The interleukin-2 receptor. *J. Biol. Chem.*, **266**, 2681–2684.

WANG, H.M. & SMITH, K.A. (1987). The interleukin 2 receptor: functional consequences of its bimolecular structure. *J. Exp. Med.*, **166**, 1055–1067.

WANG, Y.L., KANBOUR, A., HERBERMANN, R.B. & WHITESIDE, T.L. (1989). Lymphocytes infiltrating human ovarian tumours: synergy between tumour necrosis factor-α and interleukin 2 in the generation of CD8+ effectors from tumour-infiltrating lymphocytes. *Cancer Res.*, **49**, 5979–5985.

YAGITA, M., ITOH, K., TSUDO, M., OWEN SCHAUB, L.B., PLATSOUCAS, C.D., BALCH, C.M. & GRIMM, E.A. (1989). Involvement of both Tac and non-Tac interleukin 2-binding peptides in the interleukin 2-dependent proliferatin of tumour-infiltrating lymphocytes. *Cancer Res.*, **49**, 1151–1159.

ZAMBELLO, R., TRENTIN, L., PIZZOLO, G., BULIAN, P., MASCIARELLI, M., FERUGLIO, C., AGOSTINI, C., RAIMONDI, R., CHISESI, T. & SEMENZATO, G. (1990). Cell membrane expression and functional role of the p75 subunit of interleukin-2 receptor in lymphoproliferative disease of granular lymphocytes. *Blood*, **76**, 2080–2085.